# Mass Casualty Decontamination in a Chemical or Radiological/Nuclear Incident with External Contamination: Guiding Principles and Research Needs

**DOI:** 10.1371/currents.dis.9489f4c319d9105dd0f1435ca182eaa9

**Published:** 2015-11-02

**Authors:** Susan M Cibulsky, Danny Sokolowski, Marc Lafontaine, Christine Gagnon, Peter G. Blain, David Russell, Helmut Kreppel, Walter Biederbick, Takeshi Shimazu, Hisayoshi Kondo, Tomoya Saito, Jean- René Jourdain, Francois Paquet, Chunsheng Li, Makoto Akashi, Hideo Tatsuzaki, Lesley Prosser

**Affiliations:** Office of the Assistant Secretary for Preparedness and Response; Chemical Events Working Group of the Global Health Security Initiative; Chemical Emergency Preparedness and Response Unit, Health Canada, Ottawa, Canada; Chemical Events Working Group of the Global Health Security Initiative; Chemical Emergency Preparedness and Response Unit, Health Canada, Ottawa, Canada; Chemical Events Working Group of the Global Health Security Initiative; Chemical Emergency Preparedness and Response Unit, Health Canada, Ottawa, Canada; Chemical Events Working Group of the Global Health Security Initiative; 4Health Protection Research Unit in Chemical and Radiation Threats and Hazards, Medical Toxicology Centre, Newcastle University, Newcastle-upon-Tyne, U; Chemical Events Working Group of the Global Health Security Initiative; WHO-Collaborating Centre for Chemical Incidents, Public Health England, Cardiff Metropolitan University, Cardiff, Wales, UK; Chemical Events Working Group of the Global Health Security Initiative; Bundeswehr Medical Service Academy, Division of Medical NBC-Protection, German Armed Forces, Munich, Germany; Chemical Events Working Group of the Global Health Security Initiative; Strengthening Global Biosecurity, Robert Koch-Institut, Berlin, Germany; Chemical Events Working Group of the Global Health Security Initiative; Department of Traumatology and Acute Critical Medicine, Osaka University Faculty of Medicine, Osaka, Japan; Chemical Events Working Group of the Global Health Security Initiative; National Hospital Organization Disaster Medical Center, Tachikawa, Japan; Chemical Events Working Group of the Global Health Security Initiative; Department of Health Crisis Management, National Institute of Public Health, Saitama, Japan; Radiological/Nuclear Threats Working Group of the Global Health Security Initiative; Institute for Radiological Protection and Nuclear Safety, Fontenay-aux-Roses, France; Radiological/Nuclear Threats Working Group of the Global Health Security Initiative; Institute for Radiological Protection and Nuclear Safety, Saint-Paul lés Durance, France; Radiological/Nuclear Threats Working Group of the Global Health Security Initiative; Radiation Protection Bureau, Health Canada, Ottawa, Canada; Radiological/Nuclear Threats Working Group of the Global Health Security Initiative; National Institution of Radiological Sciences, Chiba, Japan; Radiological/Nuclear Threats Working Group of the Global Health Security Initiative; National Institution of Radiological Sciences, Chiba, Japan; Radiological/Nuclear Threats Working Group of the Global Health Security Initiative; Centre for Radiation, Chemical, and Environmental Hazards, Public Health England, Chilton, UK

## Abstract

Hazardous chemical, radiological, and nuclear materials threaten public health in scenarios of accidental or intentional release which can lead to external contamination of people.  Without intervention, the contamination could cause severe adverse health effects, through systemic absorption by the contaminated casualties as well as spread of contamination to other people, medical equipment, and facilities.  Timely decontamination can prevent or interrupt absorption into the body and minimize opportunities for spread of the contamination, thereby mitigating the health impact of the incident.  Although the specific physicochemical characteristics of the hazardous material(s) will determine the nature of an incident and its risks, some decontamination and medical challenges and recommended response strategies are common among chemical and radioactive material incidents.  Furthermore, the identity of the hazardous material released may not be known early in an incident.  Therefore, it may be beneficial to compare the evidence and harmonize approaches between chemical and radioactive contamination incidents.  Experts from the Global Health Security Initiative’s Chemical and Radiological/Nuclear Working Groups present here a succinct summary of guiding principles for planning and response based on current best practices, as well as research needs, to address the challenges of managing contaminated casualties in a chemical or radiological/nuclear incident.

## Background

Hazardous chemicals and radioactive materials – collectively called hazardous materials for the purpose of this paper - pose serious threats to public health by accidental release or intentional dissemination. Many such substances are present in large quantities throughout the world, as they are used in common industrial, energy-producing, household, and other processes. Terrorists have also manufactured and/or weaponized certain hazardous materials for the purpose of intentionally harming civilians. The risks are illustrated by past incidents, both accidental and intentional, such as the release of methyl isocyanate from a chemical plant in Bhopal, India in 1984^1^, the attacks by the cult Aum Shinrikyo on Japanese civilians with the nerve agent sarin in 1994 and 1995^2^, and the earthquake-induced damage and core meltdown of the Fukushima Daiichi nuclear power plant in Japan in 2011^3^. Each of these incidents caused exposure of dozens to thousands of people to hazardous material.

In this type of scenario, people can become contaminated by the hazardous material(s) through various routes of exposure, potentially leading to severe adverse health effects. External contamination may result from airborne release of a hazardous material which is then deposited on skin, eyes, hair, and/or clothing; or it may result from a person’s direct contact with a liquid or solid substance which gets transferred to skin, eyes, hair, and/or clothing. In either case, if not removed, the contaminant can be absorbed into the body through the skin or eyes and cause toxicity. Contaminant on a person’s skin, hair, or clothing also presents risks of inadvertent ingestion or re-aerosolization followed by inhalation. Once a hazardous material is ingested, inhaled, or absorbed through the skin or eyes, it is considered to be internal contamination. Decontamination – any process, method, or action that leads to a reduction, removal, neutralization, or inactivation of contamination – prevents or limits absorption of external contamination into the contaminated person’s body and also prevents transfer of the contaminant to other people and objects. Decontaminating an individual who is externally contaminated with a hazardous material, therefore, protects the individual’s health as well as the health of other community members.

Heightened concern about the risk of terrorist attacks causing mass casualties with chemical, biological, radiological, or nuclear agents, as well as initiatives to prepare for large scale chemical and nuclear accidents, have driven investments in equipment and efforts to develop capabilities for decontaminating large numbers of people in a rapid and efficient manner. For example, within the United Kingdom (UK), England’s Department of Health and equivalent structures in the devolved administrations of Northern Ireland, Scotland, and Wales purchased mobile decontamination units and distributed them throughout the country^4^. The United States (US) Hospital Preparedness Program in the early 2000s required awardees to ensure that adequate portable or fixed decontamination system capability existed statewide.

However, mass casualty decontamination methods, practices, and equipment have evolved with sparse science to shape them. Insufficient attention has been devoted to defining mass casualty decontamination goals and subsequent analysis of how to integrate decontamination into an emergency response in order to best achieve those goals. A contaminated casualty, emergency responders, and bystanders are all potentially at risk of adverse health effects from the contamination. There is an urgency to decontaminate in order to reduce a casualty’s further exposure to a hazardous material, if the substance can enter the body readily. This may have to be weighed against the highest priority demand to provide other life-saving medical treatments and supportive care. At the same time, emergency responders and hospital personnel must protect themselves from becoming contaminated. Furthermore, the most appropriate response approach will depend on the nature of the incident. For example, exposure to a gas or vapor can be limited by actions other than water-based decontamination, including moving people away from and upwind of the point of release and removing clothing. A range of exposure levels may result from a hazardous materials release; in a mass exposure incident, responders and hospital personnel will need to make decisions in order to categorize and prioritize people for decontamination, including those who do not need decontamination at all. Little evidence is available to guide the risk assessment and decision making, or triage, that are vital for achieving the goals of casualty decontamination.

Evidence is also lacking to suggest the best ways to conduct decontamination once the decision to decontaminate has been made. For example, optimal conditions for applying water-based decontamination to diverse civilian populations (including children, pregnant women, elderly, and others) contaminated with various hazardous materials using different types of equipment have not been well delineated. Circumstances in which water-based decontamination should not be conducted, such as in cold weather, when the risk of hypothermia is high, and what alternative processes should be used need to be identified. Although health outcome-based goals have been defined for radioactive contamination, they have not been established for chemical contamination. It follows that metrics for determining the effectiveness of decontamination in a chemical incident are lacking. Assessing the evidence from experimental research and actual incidents could help to identify decontamination methodologies that may be effective in both chemical and radiological incidents as well as aspects of each type of incident that require a unique approach.

Toxic chemicals and radioactive materials differ in some physical or chemical properties that translate into distinct health risks when a person is externally contaminated. Many chemicals are readily absorbed through the skin, and, within certain limits, as the amount of chemical absorbed increases, the toxic effects increase. Chemical contamination, therefore, poses an acute health risk to the contaminated person him/herself. External chemical contamination of an individual creates additional public health risks by presenting opportunities for the spread of contamination to emergency responders, hospital personnel, and health care infrastructure. By contrast, most types of radioactive material do not readily cross through the skin, with a small number of exceptions such as tritium and, to a lesser degree, radioiodine. Certain beta emitters can also be exceptions; when deposited on the skin they may cause skin burns, which can lead to systemic contamination if the beta emitters enter the body through the burned skin. Wounds or otherwise impaired skin also may allow easier entry of radioactive or chemical material into the bloodstream than intact skin. Overall, however, external radioactive contamination presents much less of an acute health risk to the contaminated individual than chemical contamination. Rather, the primary health risk associated with external radioactive contamination is that it can be internalized through ingestion or inhalation. A contaminated individual can inadvertently ingest or inhale the contaminant directly and/or spread the material to other people and objects, causing secondary exposure.

Decontamination of the contaminated individual mitigates health risks to the contaminated individual and to others to whom the contamination could spread. Yet, casualty decontamination must be integrated with other aspects of the response. The nature of the contaminant helps to determine response priorities and their urgencies. Many chemicals are quickly absorbed and begin to exert their deleterious effects within very short times, on the order of minutes to tens of minutes. Casualties may need both decontamination and medical treatment, such as administration of a therapeutic pharmaceutical or supportive care, urgently. Decision making is usually necessary at the time of the incident to choose a response strategy that is most appropriate for the situation. For known radioactive contamination, recommended response strategies are better defined and priorities have been easier to establish in pre-incident planning than for a chemical incident. This is due to the relatively low likelihood of acute adverse health effects from external radioactive contamination as well as the widespread capability among responders to measure radioactive contamination and compare it to established acceptable levels. The hazard presented by radioactive contamination is not considered significant enough to warrant a delay in the administration of life-saving medical treatment, if needed, in order for the individual to be decontaminated^5^ (International Atomic Energy Agency, 2005). However, the specific identity or even the general nature of the contamination in a hazardous materials incident may not be known immediately. Emergency preparedness and response communities may benefit from comparing the evidence and harmonizing approaches, when appropriate, between chemical and radioactive contamination incidents, forging the way for an optimised, albeit generic, approach.

Advances have been made in the science and practice of mass casualty decontamination in recent years. Research teams in Europe have conducted well designed studies, the results of which have been incorporated into best practice guidelines for European countries^6^. A group in the UK is currently funded by the US Department of Health and Human Services to continue studies of the conditions for the most effective water and soapy water-based decontamination. Hazardous Area Response Teams (HART), who can provide life-saving medical care in a hot zone or other non-permissive environments, now operate in most major cities in the UK. Similar response services are being rolled out in other regions of the UK. The French plan for responding to an urban chemical attack also provides for specially trained physicians, paramedics and firefighters to administer life-saving medical treatments before and during decontamination^7^^,^ 8 ^,^ 9. The Japanese government published official decontamination guidelines in 2004, which are regularly tested in joint civil protection exercises involving local and national governments. Germany also developed an updated concept for the decontamination of casualties exposed to hazardous materials. In 2005, the International Atomic Energy Agency and the World Health Organization published an emergency preparedness and response guide for the medical response to a nuclear or radiological emergency^5^. The US has also provided recommendations on using firefighting equipment for mass decontamination^10^ and evidence-based national planning guidance for conducting mass casualty decontamination in a chemical incident^11^. There has been consideration of strategies for both self-care decontamination (Monteith and Pearce, 2015) and responder management of the public (Carter et al., 2015) during mass exposure chemical incidents. A PubMed search reveals that in 2015, papers were also published by researchers in several countries on specific technical aspects of decontamination, such as hair 14 and wounds 15 ^,^ 16, together with studies exploring the efficacy of novel decontamination products 17 ^,^ 18 and Reactive Skin Decontamination Lotion 19.

These are examples of recent progress; however, significant knowledge and planning gaps remain. Research is needed to identify strategies for conducting decontamination of a heterogeneous civilian population that is likely to consist of the young, pregnant women, the elderly and frail, as well as a mixture of individuals of differing ethnic and cultural backgrounds, collectively contributing to susceptibility. Decontamination, therefore in a mass exposure incident, must take account of these factors to yield the best health-based outcomes. Evidence-based guidelines should be published and incorporated into plans and training curricula by local response organizations. A more intensive, concerted, and sustained effort supported by health policy and emergency preparedness decision makers is needed to address these gaps and enhance preparedness for mass casualty decontamination.

The Global Health Security Initiative (GHSI) is an informal network of countries formed in 2001 to ensure health-sector exchange and coordination of practices in confronting risks to global health posed by chemical, biological and radio-nuclear threats, as well as by pandemic influenza. The member countries/organizations of the GHSI are Canada, France, Germany, Italy, Japan, Mexico, the United Kingdom, the United States and the European Commission. The World Health Organization (WHO) is a technical advisor. As part of the GHSI partnership, an annual meeting of Health Ministers is held to foster dialogue on topical policy issues and promote collaboration. Other initiatives involving senior health officials as well as policy, technical and scientific personnel take place on a regular basis, focused on risk management; communications; chemical events; radio-nuclear threats; pandemic influenza; and global laboratory cooperation. The GHSI Chemical Events and Radiological/Nuclear Threats Working Groups, based on a series of workshops that included additional subject matter experts, have developed recommendations on the fundamental principles that should guide mass casualty decontamination in a chemical or radiological/nuclear incident and on areas of research that could drive improvements in mass decontamination effectiveness and efficiency. The scope of the work presented here is chemical and radioactive materials. Biological agents also pose important risks which may necessitate patient decontamination. In future work, these guiding principles and research needs can be built upon to address biological agents.

## Guiding Principles for Conducting Mass Casualty Decontamination in a Chemical or Radiological/Nuclear Incident

GHSI subject matter experts wish to highlight the following fundamental concepts. The principles should be applied flexibly, using expert judgment shaped by the circumstances, and according to a country or local jurisdiction’s laws, regulations, policies, and resources. For additional information, please refer to the following documents, as well as other country-specific or international guidances:

*Generic procedures for medical response during a nuclear or radiological emergency* (International Atomic Energy Agency, 2005)^5^

*Patient Decontamination in a Mass Chemical Exposure Incident: National Planning Guidance for Communities* (United States Departments of Homeland Security and Health and Human Services, 2014)^11^

*Initial clinical management of patients exposed to chemical weapons* (World Health Organization, 2014)^20^

1. Casualty decontamination is a medical[1] and public health countermeasure – it can mitigate morbidity and mortality in casualties, whilst also preventing or limiting exposure of the wider community. Casualty decontamination is one of a suite of medical and public health countermeasures to prevent, limit, or treat exposure to a hazardous material.
2. Goals of casualty decontamination should be health outcome-based and include the following (the relative importance of each of the distinct goals will depend on the situation, including details such as the type of contamination, number of casualties, route of exposure, and environmental conditions): reduce the probability of harm to the casualty; protect the health of responders and receivers; protect the wider community; prevent contamination of health care equipment and facilities and thereby maintain the function of the health care system to treat all patients; and ease the concerns of individuals who feel they may have been exposed.
3. Casualty decontamination must be well coordinated with the medical aspects of the incident response in order to maximize the potential for achieving the above mentioned goals. Decisions about whether to decontaminate or not and how to decontaminate should be a component of the triage phase of the response, and take into consideration the medical status of the casualty. Evaluation of the need for life-saving treatment should be followed quickly by decisions on decontamination. Decontamination should not delay other urgent medical treatment. In the case of radioactive contamination, life-saving treatments should take priority over decontamination^5^ . When facing chemically contaminated casualties, responders and hospital personnel must assign relative priorities to life-saving medical treatments and decontamination using risk-based judgment of factors including the magnitude and type of contamination, medical status, and available resources. If deemed necessary, decontamination for chemicals should be performed as early as possible, since many chemicals are readily absorbed into the body and act quickly. Similarly, early decontamination for radioactive materials, especially when open wounds are present, can help reduce the individual’s exposure and prevent incorporation of radionuclides into the body. A similar approach should be considered for chemical-contaminated wounds. Decisions about when decontamination is sufficient should take into account a variety of information sources, including clinical judgment, detector sampling results from casualties and the environment, modeling predictions, on the ground situation, and any other pertinent factors. While radioactive contamination on skin can be directly measured, similar technologies may not be available or practical for a chemical exposure incident; clinical judgment, including diagnostic markers, and other information about the incident must be relied upon in cases of chemical contamination.
4. Decontamination should not harm the casualty. The potential for adverse effects such as hypothermia should be recognized. Decontamination methods themselves must not promote absorption of the contaminant through skin into the body, spread the contaminant to additional areas of skin, or aerosolize the contaminant so that it can be inhaled.
5. Containing the contaminant as close as possible to the point of release by limiting access to and limiting the spread of contaminant from contaminated areas is essential for achieving health outcome-based goals.
6. Hospitals should be prepared to receive potentially contaminated patients. Past incidents demonstrate that a significant proportion of potentially contaminated individuals may leave the scene and arrive at hospitals without having been evaluated, decontaminated, treated, or transported by responders. Like first response organizations, hospitals must have the capabilities to evaluate the need for and conduct decontamination, in conjunction with medical evaluation and treatment, as well as protect their staff and infrastructure from secondary exposure and/or contamination.
7. Casualty decontamination is the responsibility of local response organizations within the affected community, since outside assistance may not arrive for many hours. Local responders, at the scene and at receiving hospitals, should have appropriate personal protective equipment and be trained in its proper use.
8. Coordination among the various response organizations within the affected community is essential.
9. A risk communication strategy to address the public, covering those who were at the incident and those were not, should be established during planning and preparedness activities^21^. Instructions on self-decontamination would describe actions that individuals can execute themselves, without the assistance of responders. These include moving away from and upwind of the point of hazardous material release, removing at least outer layers of clothing, wiping off visible contamination, and using clean water to rinse contaminated areas of skin.

[1]* Medical countermeasures include both pharmaceutical interventions, such as vaccines, antimicrobials, antidotes, and antitoxins, and non-pharmaceutical interventions, such as ventilators, diagnostics, personal protective equipment (PPE), and patient decontamination that may be used to prevent, mitigate, or treat the adverse health effects of an intentional, accidental or naturally occurring public health emergency* 22. *In some settings, casualty decontamination is characterized as a protective action, which is encompassed by the broad definition of medical countermeasure cited here, as it can prevent adverse health effects in the exposed individual as well as in other people to whom the contamination could spread.*

## Priority Research Needs

Decontamination of casualties in a mass exposure incident has only recently begun to gain wide acceptance as a medical and public health countermeasure; thus, many questions about its optimal utilization have not been explored. New research and analysis would allow more evidence-based mass casualty decontamination practices to be established 11 ^,^ 23 ^,^ 24 ^,^ 25 ^,^ 26. Three broad topics have been identified by GHSI subject matter experts as priority targets for rigorous scientific investigation.

**Operational analysis to determine the best ways to integrate casualty decontamination into the incident response and coordinate it with medical evaluation and treatment.** Various ways of organizing a response to incorporate mass casualty decontamination have not been compared for their efficacy in mitigating morbidity and mortality. Evaluation of exercises and training, as well as other types of operational analysis and research could lead to better understanding of the most effective ways to achieve such integration. Decision support tools are needed to help guide decisions during triage on if, when, and how to decontaminate casualties, and how decontamination will be coordinated with medical evaluation and treatment. The logistical challenges of having medical countermeasures available and responders administering them in the field while wearing appropriate personal protective equipment should be included. This work should also take into account country, region, state, and other locality specific laws, regulations, organizational structures, practices, and other considerations that could limit the generalizability of mass casualty decontamination strategies.

**Comparative study of the efficacy of various decontamination methods and their potential adverse effects.** Limited research has been conducted on technical casualty decontamination methods, such as the optimal parameters for water-based decontamination. A US-funded project currently underway by UK researchers is assessing the effects of certain variables, such as water flow rate, temperature, and detergent type, on decontamination efficacy and adverse effects using a firefighting equipment-based decontamination system. This work will need to be extended and expanded with future studies, since many factors contribute to the way that water-based decontamination is delivered. An extensive body of work may be required to identify optimal parameters for maximizing efficacy and minimizing adverse effects. Water and soap have been demonstrated to effectively decontaminate skin contaminated with radionuclides.

**Behavioral, communication and privacy issues: what do casualties and community members need and how to meet those needs in ways that will best support compliance with and effectiveness of casualty decontamination.** Various communication, behavioral and social factors can significantly influence people’s actions during a disaster, yet strategies for using these factors to optimize compliance with and effectiveness of decontamination have not been thoroughly studied. UK researchers have begun to investigate the effects of various types of instructions provided to casualties undergoing decontamination 27, while Canada has considered how to provide psychosocial support to people who need decontamination during a radiological, nuclear, or chemical emergency 28. However, as with technical decontamination methods, communication is shaped by multiple factors and plays a role throughout the decontamination process. Enhanced decontamination effectiveness depends on coupled study of behavioral, communication, and privacy issues with technical methods and requires additional attention.

## Conclusion

The GHSI Chemical Events and Radiological/Nuclear Threat Working Groups have identified current challenges to effective casualty decontamination in a mass exposure incident. Application of the guiding principles described here to preparedness, planning, and response activities will help to improve effectiveness of decontamination as well as other aspects of the medical management of casualties in a mass exposure incident, based on our current scientific understanding. Research programs designed to address the priority research needs will strengthen the scientific evidence, upon which further improvements in preparedness, planning and response can be based.

## Competing Interests

The authors have declared that no competing interests exist.
